# Steric and Stereochemical Modulation in Pyridyl- and Quinolyl-Containing Ligands

**DOI:** 10.3390/molecules21121647

**Published:** 2016-12-01

**Authors:** Zhaohua Dai

**Affiliations:** Department of Chemistry and Physical Sciences, Forensic Science Program, Pace University, 1 Pace Plaza, New York, NY 10038, USA; zdai@pace.edu; Tel.: +1-212-346-1760

**Keywords:** pyridine, quinoline, ionophore, sensor, steric effect, stereochemical control, metal ions, chiroptical, selectivity, differentiation

## Abstract

Nitrogen-containing pyridine and quinoline are outstanding platforms on which excellent ionophores and sensors for metal ions can be built. Steric and stereochemical effects can be used to modulate the affinity and selectivity of such ligands toward different metal ions on the coordination chemistry front. On the signal transduction front, such effects can also be used to modulate optical responses of these ligands in metal sensing systems. In this review, steric modulation of achiral ligands and stereochemical modulation in chiral ligands, especially ionophores and sensors for zinc, copper, silver, and mercury, are examined using published structural and spectral data. Although it might be more challenging to construct chiral ligands than achiral ones, isotropic and anisotropic absorption signals from a single chiroptical fluorescent sensor provide not only detection but also differentiation of multiple analytes with high selectivity.

## 1. Introduction

As nitrogen-containing aromatic compounds, pyridine and quinoline can form complexes with many metal ions because the lone pair electrons on the nitrogen are available for coordination since they are not part of the aromatic systems. The aromatic ring of pyridine or quinoline itself is a rigid platform, which can be incorporated into many achiral and chiral binding pockets to build ligands of different affinities to different metal ions. Chiral pyridyl- or quinolyl-containing ligands and their metal complexes are used as catalysts in asymmetric catalysis [1]. A recent review described the design principle for selective metal ion binding and sensing using many achiral pyridyl-containing ligands [2]. There are some other examples of achiral pyridyl-containing ligands used for metal sensing and this article will discuss the structural features of some of them [3,4,5,6]. This article mainly focuses on the stereochemical approach to achieving selective metal binding and sensing using pyridyl-/quinolyl containing ligands, especially chiral ligands. The structure-activity relationship in the modulation of coordination chemistry and/or signal transduction using such ligands/sensors will be discussed.

## 2. Steric and Stereochemical Modulation of Binding Affinity and Selectivity

According to Comba [7], the design of a selective ligand for a metal ion must involve a high degree of preorganization for the specific metal ion and also a high degree of “disorganization” or mismatch for competing metal ions. The latter is not an easy task and it has not been addressed in detail. Still et al*.* pointed out that an important principle in the rational design of synthetic host molecules is using substitution and stereochemistry to reduce the populations of conformations unfavorable to binding [8]. By the same token, substitution and stereochemistry manipulation should be able to reduce the population of conformations favorable to binding, thus enabling the manipulation of selectivity.

### 2.1. Steric Control of Achiral Ligands’ Selectivity to Zn^2+^ and Cu^2+^

Many ionophores are incorporated in sensors for metal ions and other species. Signal transduction of many sensing events depends on the structure and conformation of the analyte-sensor complexes. In the development of Zn^2+^ chelators and sensors, specificity to zinc is highly desirable from many perspectives. Over the years, some sensitive fluorescent sensors for Zn^2+^, which are mostly N-containing ligands, have been developed and their selectivity against some other metal ions has been investigated [6,9,10,11,12,13,14,15,16,17,18,19,20,21]. However, as predicted by the Irving-Williams series [22], Cu^2+^ complexes with nitrogen donor ligands are typically found to be more stable than Zn^2+^ complexes by several orders of magnitude: Complexation with d^10^ metal ion Zn^2+^ offers no ligand field stabilization energy (LFSE) [23]. For example, macrocycles are ideal for the selective coordination of alkali or alkali earth metals, which can be discriminated from each other solely on the basis of their ionic radii and charge. However, they may be less useful in distinguishing Cu^2+^ from Zn^2+^ because the radii of these ions are almost identical. This is reflected in the relative binding affinities of macrocyclic ligands toward Zn^2+^ and Cu^2+^, where the latter is favored by 10–15 orders of magnitude [21,24].

One simple Schiff base sensor exhibits Zn^2+^-chelation enhanced fluorescence, which suffers from interference from Cu^2+^. A subtle structural change can turn such a ligand platform from an enol-imine tautomer to a keto-enamine tautomer, which is much more selectively for Zn^2+^ over Cu^2+^ [25].

**Figure 1:**
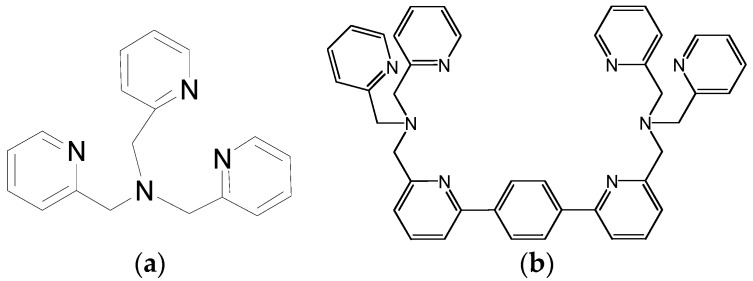
Structures of (**a**) compound **1**, tris(2-pyridylmethyl)amine (TPA) and (**b**) compound **2**, parabenzobis-TPA (PBTPA).

Rorabacher et al*.* examined the steric effect on Cu^I/II^ redox couples [26]. They found that in aqueous solution for 35 different tripodal ligands, many of which are tris(2-pyridylmethyl)amine (TPA, Figure 1) analogs with different substituents, the stability constants of their Cu^+^ complexes are in the relatively narrow range of 10^12^–10^16^ although they are hugely different in terms of coordination geometry and donor strength, while those of their Cu^2+^ complexes stretch over 26 orders of magnitude. It was suggested that ligand coordination geometry mainly impacts the complexation of Cu^2+^, while imposing little effect on Cu^+^.

**Figure 2:**
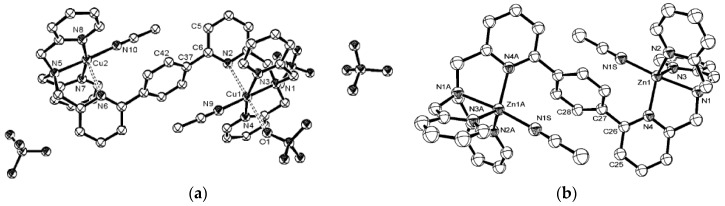
X-ray crystal structure of the Zn^2+^ (**a**) and Cu^2+^ (**b**) complexes of PBTPA. Reprinted with permission from Reference [28]. Copyright (2003) American Chemical Society.

Because of its 3d^10^4s^0^ configuration, Zn^2+^, like Cu^+^ [22], is not strongly influenced by constraints in its coordination configuration. Therefore, it is necessary to make a ligand which has a high degree of predisorganization for Cu^2+^ so that it can show better Zn^2+^/Cu^2+^ selectivity. As a d^9^ metal, the bonding in Cu^2+^ complexes is partially covalent. At the same time, Cu^2+^ prefers 4-coordinate square planar and 5-coordinate square pyramidal geometries over tetrahedral and trigonal bipyramidal geometries according to Crystal Field Theory [27]. *C*_3_ or *pseudo-C*_3_ symmetrical N-containing TPA derivatives bind Cu^2+^ much better than Zn^2+^ as predicted by the Irving-Williams series [22]. At the same time, they are naturally trigonal bipyramidal geometry providers and all of their Zn^2+^ complexes are of trigonal bipyramidal configuration to the author’s awareness. To accommodate Cu^2+^, this trigonal bipyramidal configuration is often distorted to resemble a square (bi)pyramidal. This preference is clearly demonstrated in parabenzobis-TPA (PBTPA, Figure 1) [28], one of the tripodal ligands based on TPA. X-ray structures (Figure 2) shows that it forms a trigonal bipyramidal complex with zinc (with N_Py_-Zn-N_Py_ bond angles of 117.3°, 114.9°, and 118.5°) and a square bipyramidal complex with copper(II) (with N_Py_-Cu-N_Py_ bond angles of 85.2°, 95.6°, and 164.4°). Some conformationally mobile chiral TPA analogs also form square pyramidal complexes with Cu^2+^ [29]. To accommodate other metals such as Fe^3+^ [30] and V^4+^ [31] complexes, such distortion is also needed.

**Figure 3:**
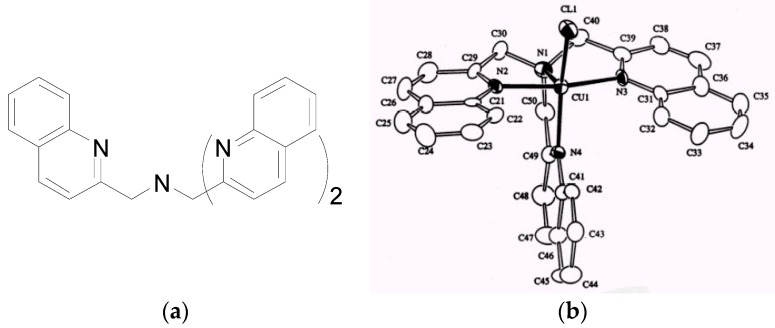
(**a**) Tris(2-quinolylmethyl)amine (TQA) and (**b**) X-ray structure of a TQA-Cu^2+^ complex. Reprinted with permission from Reference [32]. Copyright (1994) American Chemical Society.

Karlin et al*.* found that tris(2-quinolylmethyl)amine (TQA) (Figure 3) and its analogs form *C_σ_* symmetrical square pyramidal complexes with Cu^2+^ (Figure 3) [32]. Other researchers found that steric overcrowding decreases the formation constants between TPA/TQA type of ligands and metal ions. The extent of such a decrease is inversely proportional to the ion sizes. Significant decrease is seen with small ions such as Zn^2+^, while smaller decrease is seen with larger ions such as Pb^2+^ because the Zn-N bond length is distorted in the Zn^2+^-complex with overcrowded ligands [33].

**Figure 4:**
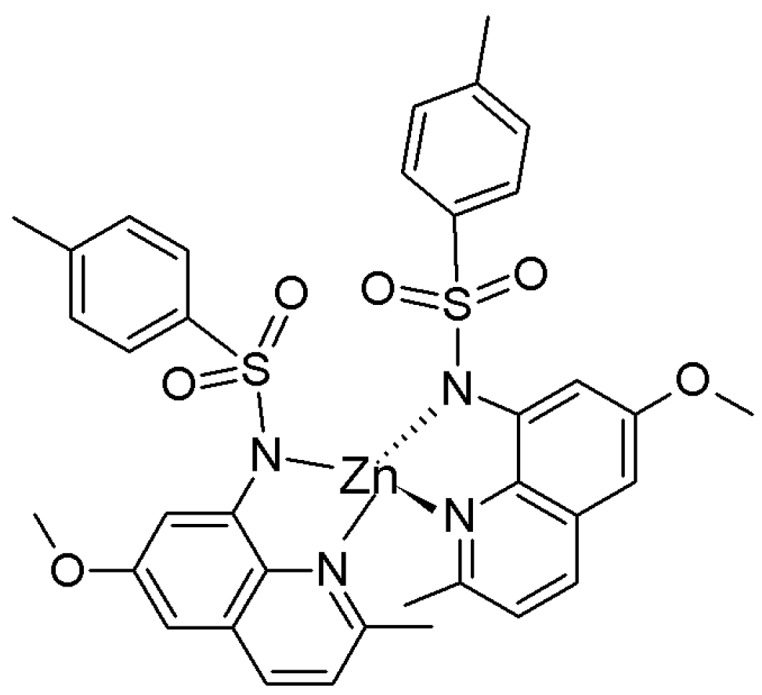
The Zn^2+^ complex of 2-Me-TSQ.

One of the most widely used fluorescent zinc sensors is TSQ [34,35]. Its analogs Zinquin and 2-Me-TSQ (Figure 4) show better Zn^2+^/Cu^2+^ selectivity than most other fluorescent probes, although they still bind Cu^2+^ stronger than Zn^2+^ [36,37]. The overall binding constant of the Cu^2+^ complex (logβ_2_ = 18.3 ± 0.05) of Zinquin is only slightly greater than that of its Zn^2+^ counterpart (logβ_2_ = 17.54) [12]. A complex between Zn^2+^ and 2-Me-TSQ can be formed with a 1:2 stoichiometry (Figure 4). The methyl groups at the 2-position of each of the two quinolones would clash with each other in a square planar or an octahedral complex, although the bite angle of 83° accommodates these two geometries. As a result, a distorted tetrahedral geometry is formed to relieve such steric hindrance, as shown in its X-ray crystal structure (not shown in Figure 4) [37]. However, it may be energetically less favorable for Cu^2+^, which might account for the lower than expected affinity for Cu^2+^. Therefore, engineering a ligand scaffold that can exert significant steric restrictions upon metal coordination geometry appears to be a promising approach for the design of Zn(II)-selective fluorescence probes.

### 2.2. Stereochemical Control of Chiral Ligands’ Zn^2+^/Cu^2+^ Selectivity

Although it sounds outlandish to modulate the behavior of achiral metal ions through chiral organic ligands, it is not without precedence. Mother Nature utilizes chiral substances in non-asymmetric processes. Lasalocid is a natural ionophore for Na^+^ with several asymmetric carbons in its skeleton. The metal-binding functions of its stereoisomers depend on their stereochemistry, as do those of some tetrahydropyranoid podand ionophores [8,38,39]. Similar results have been found in another polyether ionophore and metal-binding antibiotic, monensin, its stereoisomers, and analogs [40]. Ca^2+^ is selectively bound by an isomer of the hydroxylated-bistetrahydrofuran skeleton of a potent antitumor agent, *annonaceous* *acetogenins*, against other alkali or alkali-earth metal ions [41].

**Figure 5:**
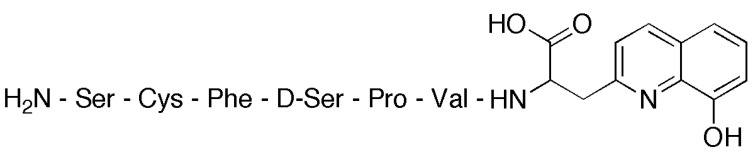
Fluorescent zinc sensor containing 8-HQ and minimized, a zinc finger protein-based peptide sequence.

Zinc finger peptides bind zinc exceptionally well, with dissociation constants as low as 5.7 pM [42], because they possess peptidyl domains 25–30 residues in length that form pre-organized metal binding pockets highly selective for divalent zinc. Imperiali et al. explored the “hybrid approach” by constructing and attaching peptidyl domains seven residues in length to the 8-HQ fluorophore, which is structurally similar to TSQ. The peptidyl domain was carefully designed to try to preserve the architecture of the zinc finger motif using minimized size and complexity. One of the reported ligands is shown in Figure 5 [43]. The cysteine residue is key to enhancing the zinc selectivity of the ligand.

The (*R*,*R*)- or (*S*,*S*)-isomer of a bis-chiral crown ether has been reported to bind selectively sodium against potassium due to the optimized size of the binding pocket, while its optically inactive (*R*,*S*)-isomer does not show such selectivity [44].

Balaz et al*.* used single-labeled pyridylporphyrin–DNA conjugates to sensitively and selectively detect Hg^2+^ in water, although pyridylporphyrin rather than the nucleobase was found to play a crucial role in Hg^2+^ binding and sensing [45].

**Figure 6:**
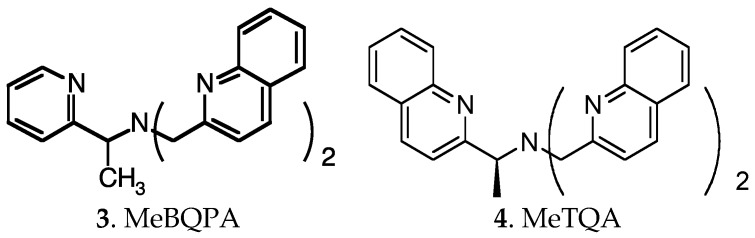
Structures of chiral TPA/TQA derivatives MeBQPA and MeTQA.

In a study by Castagnetto et al*.* recognition of Zn^2+^ by MeBQPA (Figure 6), a chiral derivative of TPA/TQA, benefited from both fluorescence enhancement as well as chiroptical signal increase [46]. However, Cu^2+^ was a significant competitor for Zn^2+^ in that system, as it is for all ligands based on the TPA scaffold [47]. In solution, Canary et al*.* found that the chiral TQA derivative MeTQA (Figure 6) forms Cu^2+^ complexes with geometries between square pyramidal and trigonal bipyramidal, in some cases even predominantly square pyramidal, as revealed by UV [48].

**Figure 7:**
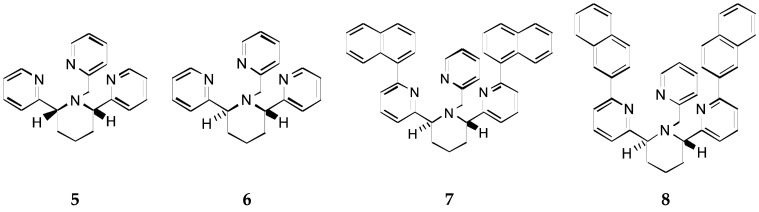
Structures of piperidine derivatives of TPA.

To improve Zn^2+^/Cu^2+^ selectivity, further preorganization to impose a trigonal bipyramidal coordination geometry is needed. Rigidification is a common approach to preorganization, and in principle it should also work in predisorganization. The orientation and interaction between substitution groups can also exert influences on selectivity as shown in Zinquin complexes. Therefore, one can envision that it is possible to construct a ligand whose structure is chirally synchronized and mechanically rigidified so that its trigonal pyramidal configuration cannot be bent to a planar geometry. Such manipulation would depress its Cu^2+^ affinity, while exerting much smaller compromise, if any, on its Zn^2+^ affinity. In Rorabacher’s words [26], one can construct tripodal ligands which are incapable of adapting to a planar geometry but could readily accommodate tetrahedral or distorted tetrahedral geometries. In this way, the higher affinity of TPA/TQA derivatives to Cu^2+^ over Zn^2+^ might be reversed. Toward this end, a stereochemical control approach was developed to engineer improved Zn^2+^/Cu^2+^ selectivity through controlling ligand stereochemistry: a ring was incorporated into the TPA ligand by connecting two of the arms to give compounds **5** and **6** (Figure 7), in which the piper dine ring reduces conformational mobility by rigidifying the compounds and two chiral centers are introduced to further control the stereochemistry [49].

For the cis-piperidine derivative **5**, Cu^2+^ and Zn^2+^ complexation gave logβ = 14.8 and 10.1, respectively, and for *trans*-ligand **6** the numbers were found to be 12.0 and 11.2, respectively [49,50]. The parent compound TPA shows logβ = 16.15 for its Cu^2+^ complex compared to 11.00 for its Zn^2+^ complex. Thus, the ratio of the association constants for the binding of Cu^2+^ over Zn^2+^ for TPA, **5**, and **6** is 1.4 × 10^5^, 5 × 10^4^, and 6, respectively. As a pair of diastereomers, piperidine compounds **5** and **6** are expected to exhibit somewhat different affinities toward metal ions. However, the difference here is so large mainly because the *trans*-piperidine ligand **6** enforces a *C_3_* coordination environment through the identical stereochemistry at the two chiral centers and the rigid piperidine ring, which makes it less favorable to Cu^2+^ binding. This qualitatively explains its dramatically improved Zn^2+^/Cu^2+^ selectivity.

Semi-empirical calculations and X-ray structures show greater similarity of the [Cu(TPA)Cl]^+^ Cu-N bond lengths in the complex with **5** than in **6** [49]. Thus, *trans*-ligand **6** appears to distort the coordination sphere of the Cu^2+^ ion, resulting in a less stable complex, while ligand **5** is preferred significantly for Cu^2+^. This agrees with the observation that the binding of Cu^2+^ is quite dependent on ligand stereochemistry while that of Zn^2+^ is not.

The *trans*-ligand **6** was tagged with naphthalene fluorophores to prepare ligands **7** [49] and **8** [51,52] (Figure 7). The fluorescence of the naphthalene moieties is diminished by photo-induced electron transfer (PET) in the absence of metal ion, but increases nearly 20-fold upon binding Zn^2+^ for compound **7**. The sensitivity of compound **8** for Zn^2+^ was found to be nanomolar in 4-(2-hydroxyethyl)-1-pieperazineethanesulfonic acid(HEPES) buffer with 1% methanol at physiological pH. The improved selectivity that had been found for the *trans* chiral piperidine scaffold was also preserved with compounds **7** and **8**. Thus, stereochemical engineering of ligands by constructing an unfriendly environment for Cu^2+^ to depress their Cu^2+^ affinity and enhance their Zn^2+^/Cu^2+^ selectivity has proven to be feasible.

### 2.3. Steric and Stereochemical Control of Ligands’ Selectivity to Ag^+^

**Figure 8:**
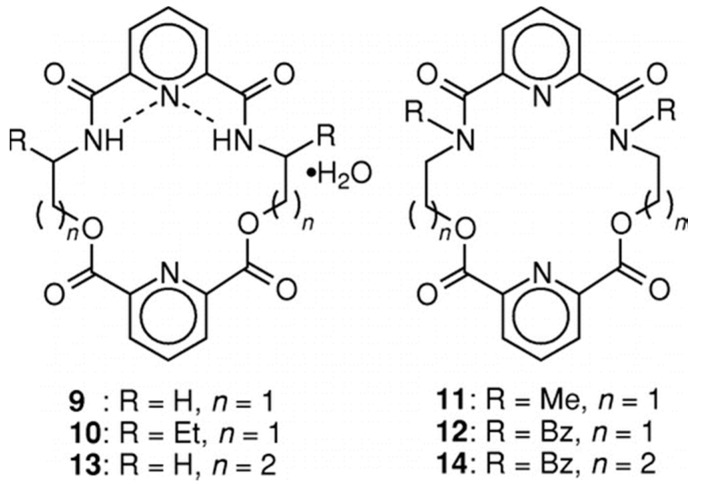
Pyridyl-containing macrocyle ligands for Ag^+^. Reprinted with permission from Reference [53]. Copyright (1996) American Chemical Society.

Some pyridyl-containing macrocycles (Figure 8) are used as ionophores for Ag^+^. Their structures and conformations affect their affinities to ions. Compounds **9**, **10**, and **13** are somewhat planar. Since there is intramolecular N_Py_-HN-amide hydrogen bonding in these free ligands, each of their cavities has to undergo significant conformational changes during complexation, resulting in poor affinity to Ag^+^. However, since the steric requirement of methyl/benzyl substituents on amide N in **11** and 1**2** takes themselves out of the cavities and positions the amide C=O toward their respective cavities, they may undergo fewer conformational changes during complexation, thus showing higher affinities to Ag^+^ over Pb^2+^, Tl^+^, alkali, and alkaline earth cations. The increased spacer length in **14** removes such steric arrangement, resulting in poor selectivity [53].

**Figure 9:**
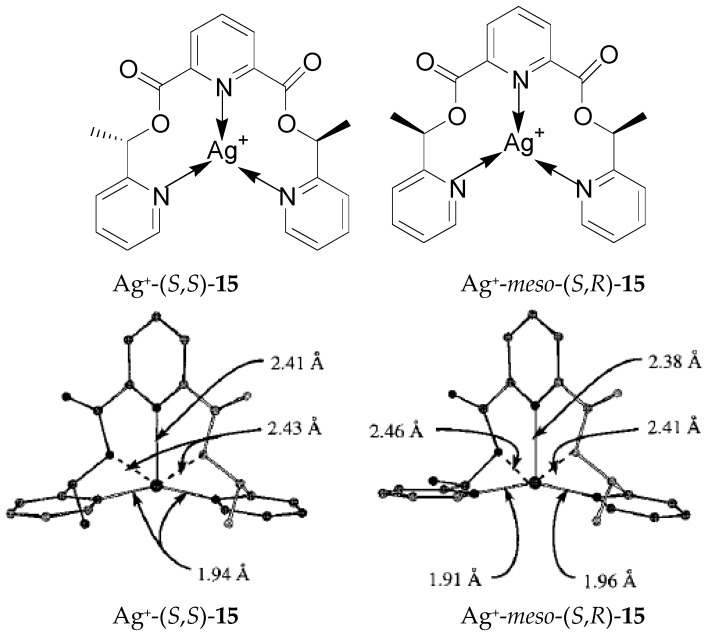
Ag^+^ complexes of ligands whose Ag^+^-specificities are controlled by stereochemistry and optimized structures of such complexes. Reprinted with permission from Reference [54]. Copyright (1998) American Chemical Society.

Proper handling of ligand stereochemistry can lead to improved Ag^+^ affinity in some podand ligands bearing pyridine moieties and two chiral arms (Figure 9, top) [54,55]. In principle, as a pair of diastereomers, (*S*,*S*)- and (*R*,*S*)-**15**, respectively, can exhibit different binding abilities toward cations. The (*S*,*S*) ligands can extract Ag^+^ more selectively and more slowly in the presence of Pb^2+^, Cu^2+^, Ni^2+^, Co^2+^, and Zn^2+^ than their corresponding *meso* ligands. Computer modeling and energy calculations showed that in the optimized structures of their Ag^+^ complexes (Figure 9, bottom), (*S*,*S*)-**15** has a symmetrical arrangement of three pyridine rings for the binding of the Ag^+^ ion, which is equally coordinated by two terminal pyridine rings, while the two terminal pyridine nitrogen atoms coordinate with the Ag^+^ ion in an asymmetrical fashion in the Ag^+^ complex with (*S*,*R*)-**15**. The energy calculations indicate that the Ag^+^-(*S*,*S*)-**15** complex is more stable by 3.46 kcal/mol than the Ag^+^-(*S*,*R*)-**15** complex. Such differences are also observed in the derivatives of these two diastereomers. It was concluded that a combination of ligand geometry and stereo-controlled substitution can improve Ag-specificity in this class of ligands [54,55].

## 3. Steric and Stereochemical Modulation of Signal Transduction

Many ionophores are incorporated in sensors for metal ions and other species. Signal transduction of many sensing events depends on the structure and conformation of the analyte-sensor complexes.

### 3.1. Modulating Fluorescence or Luminescence

**Figure 10:**
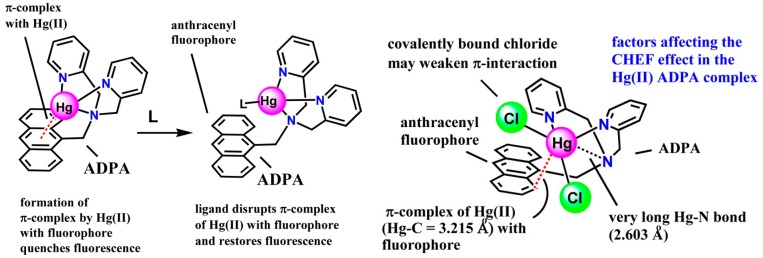
Effect of co-valent donor species on the fluorescence Hg^2+^/*N-*(9-anthracenylmethyl)-*N*-(2-pyridylmethyl)-2-pyridinemethanamine (ADPA) complex. Reprinted with permission from Reference [60]. Copyright (2012) American Chemical Society.

Many metal ions sensors employ fluorescence as their signal output. Generally “switch-on” fluorescent sensors are preferred to “switch-off” ones. There have been significant endeavors to make fluorescent sensors for Hg^2+^. Although a number of reversible “switch-on” fluorescent sensors for Hg^2+^ have been reported [56,57,58,59], many other sensors exhibit fluorescence “switch-off” upon binding Hg^2+^, which as a heavy metal turns to quench its sensors’ fluorescence through spin-orbit coupling. Hancock et al recently shed some light on the structural requirements for Hg^2+^ sensors that exhibit chelation enhanced fluorescence (CHEF) when their photo-induced electron transfer (PET) processes are handicapped by Hg^2+^ [60]. The secret is that the formation of a π-complex between the heavy metal and the fluorophore needs to be disrupted or eliminated. By examining some pyridyl-containing sensor, including *N-*(9-anthracenylmethyl)-*N*-(2-pyridylmethyl)-2-pyridinemethanamine (ADPA) (Figure 10), and other nitrogen-containing sensors from a few research groups, it is generalized that the Hg^2+^ ion should be held far enough away from the fluorophore, or covalently binding donor atoms, such as S and Br, need to be employed to limit the strength of interaction between the Hg^2+^ ion and the fluorophore. This might be able to account for the “switch-on” fluorescence of chiroptical mercury sensors that contain quinolyl and methionine/*S*-methylcysteine moieties made by the author of this article [61].

**Figure 11:**
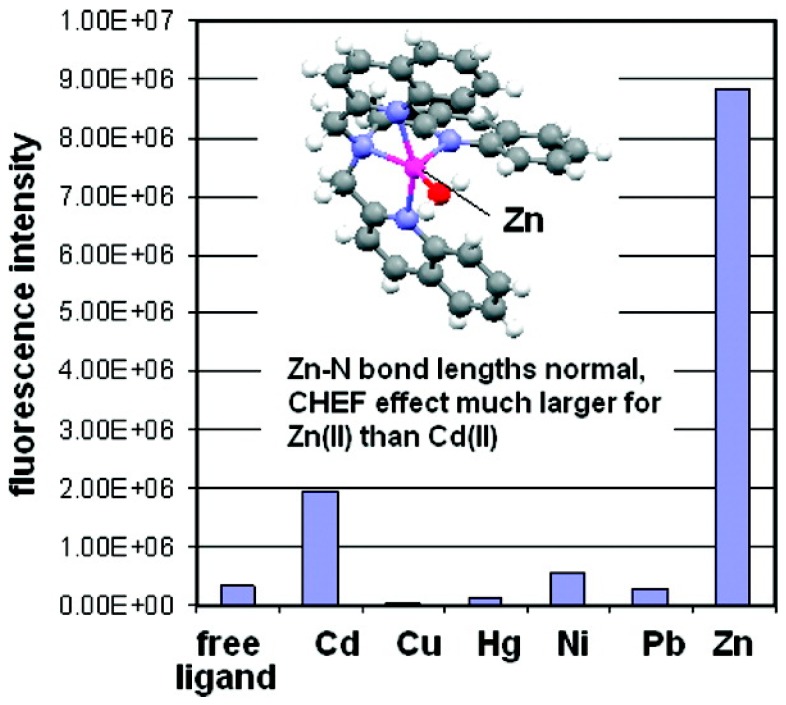
In the Zn^2+^-TQA complex, the ligand is not overcrowded and the Zn-N bond lengths are not distorted. Therefore, the CHEF effect induced by Zn^2+^ is stronger that induced by bigger ions. Reprinted with permission from Reference [33]. Copyright (2009) American Chemical Society.

Steric crowding in ligands can compromise the (CHEF) effect by small metal ions such as Zn^2+^ ion as compared to larger ions such as Cd^2+^ ion [33]. Steric crowding distorts the Zn-N bond length, which allows some quenching of fluorescence by the PET mechanism. The steric crowding increases in the following sequence for tripodal pyridyl-/quinolyl-containing ligands: TPA < TQA < tris(6-methyl-2-pyridyl)amine (TMPA). In a complex formed between Zn^2+^ and TQA, X-ray crystallography shows that the Zn-N bonds are all of normal lengths (Figure 11), which means the level of steric crowding in TQA is not severe enough to cause significant Zn-N bond length distortion. As a result, there is larger enhancement of TQA fluorescence by Zn^2+^ than by Cd^2+^, in contrast to similar but more sterically crowded TMPA where Cd^2+^ induced CHEF effect is stronger. The CHEF effect for TQA increases with the decrease in metal ions sizes.

**Figure 12:**
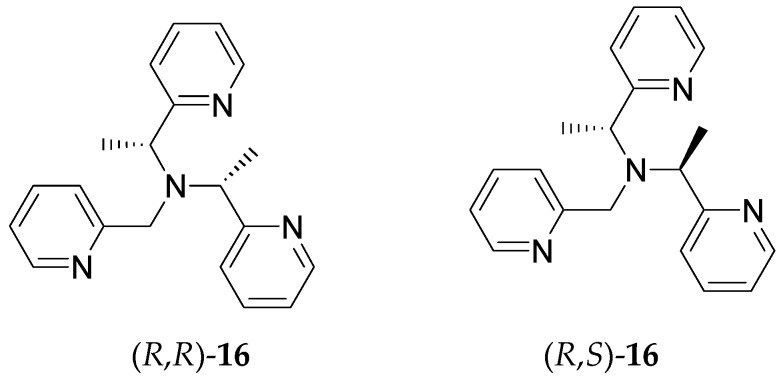
Bis-chiral TPA-analogs that can modulate lanthanide fluorescence.

Open chain TPA analogs with two chiral arms (Figure 12) have been used to tune lanthanide luminescence [62]. Lanthanide complexes formed with (*R*,*S*)-**16** ligand give stronger fluorescence than their corresponding cousins formed with (*R*,*R*)-**16** ligand. Lanthanide complexes of another pyridyl-containing ligand with two chiral centers also exhibited interesting properties in luminescence [63].

More recently, a chiral pyridyl/quinolyl-containing tripodal ligand was demonstrated to form a series of lanthanide complexes exhibiting multiple anion-sensing profiles, which can be explained by the presence of a fluorescent quinoline and a stereocontrolled methyl group resulting in differences in fluorescence, CD, and Ln(III)-luminescence signals of the anion-bound complex, which are controlled by the nature of the targeted anions [64]. It is more specific than regular fluorescence sensing.

### 3.2. Modulating Chiroptical Signals

**Figure 13:**
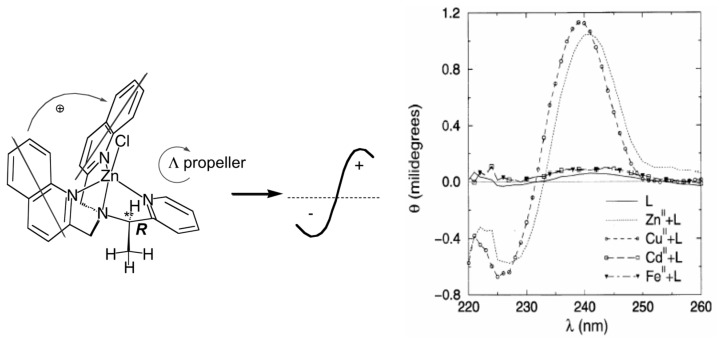
Circular dichroism spectra of (*R*)-MeBQPA and complexes with Zn(ClO_4_)_2_, Cd(NO_3_)_2_, Cu(ClO_4_)_2_, and FeCl_2_ in aqueous 4-(2-hydroxyethyl)-1-pieperazineethanesulfonic acid (HEPES) buffer. Adapted with permission from Reference [46]. Copyright (1998) the Royal Society of Chemistry.

Compared with achiral ligands, chiral ones can yield additional spectroscopic information such as chiroptical signals [65]. Zinc greatly enhances the fluorescence of MeBQPA [46], while other metal ions induce fluorescence responses. More interestingly, the ligand generates strong signals in exciton-coupled circular dichroism (ECCD) upon formation of complexes with some metal ions (Zn^2+^, Cu^2+^), while complexes with octahedral metal ions (Cd^2+^, Fe^2+^) do not give strong CD signals (Figure 13). Both isotropic (fluorescence) and anisotropic absorption (CD) signals from the optical response of this single chiral ligand are employed to provide not only detection but also differentiation of multiple analytes: Zn^2+^ (strong fluorescence and ECCD response), Cu^2+^ (strong ECCD but no fluorescence), Cd^2+^ (strong fluorescence but no ECCD) and Fe^2+^ (neither fluorescence nor ECCD).

Metal ion detection by a multimode switchable chiroptical fluorescent sensor containing both *S*-methylcysteine and quinoline moieties through both fluorescence enhancement and anisotropic absorption distinguish even more metal ions [61]. We will discuss these sensors in more detail in the “Modulating Switchable Binding Pockets in One Ligand” section.

**Figure 14:**
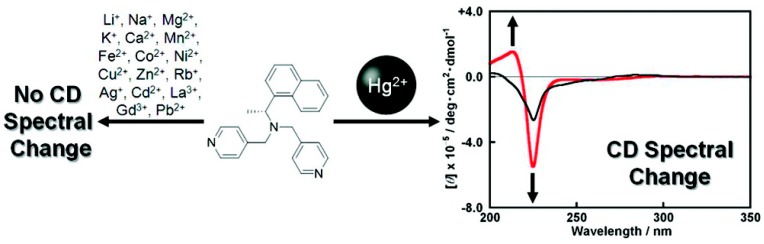
Hg^2+^ induces circular dichroism (CD) changes in a chiral sensor while many other metal ions do not. Reprinted with permission from Reference [66]. Copyright (2012) American Chemical Society.

A pyridyl-containing bidentate Hg^2+^ sensor with a chiral-center near its naphthalene chromophore has been reported more recently (Figure 14). The sensor exhibits significant CD changes when titrated with Hg^2+^, while many other metal ions do not induce such changes. Cold ESI-MS and ^1^HNMR data suggest that Hg^2+^ and the ligand form a 1:1 coordination polymer, which gives a negative exciton coupled CD (ECCD) signal, suggesting that the naphthalene units in the coordination polymer are arranged in a counterclockwise screw sense in solution. The complex’s X-ray crystallography indeed shows a polymer-like structure, although the naphthalene units in the solid state are arranged in an ECCD-inactive eclipsed form, likely caused by a packing effect [66].

There are other chiroptical sensors for metal ions that show changes in CD. However, they do not contain pyridyl or quinolyl groups [67,68,69] and will not be discussed in further detail.

**Figure 15:**
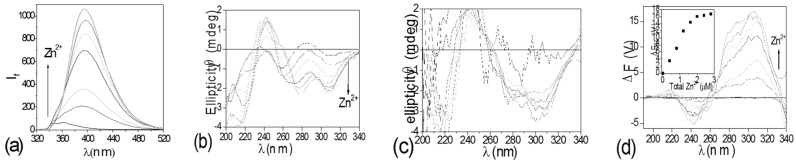
Spectral response of 2 µM (*S*,*S*)-**9** to Zn^2+^ in acetonitrile. (**a**) Fluorescence (Ex: 300 nm); (**b**) CD; (**c**) Fluorescence-detected circular dichroism (FDCD); (**d**) ΔF, inset: titration curve of 2 μM (*S*,*S*)-**9** with Zn(ClO_4_)_2_. Reprinted with permission from Reference [52]. Copyright (2004) American Chemical Society.

**Figure 16:**
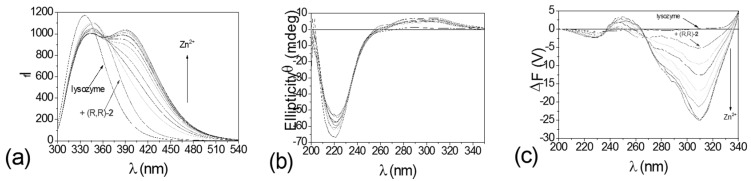
Spectral responses of 3.2 µM (*R*,*R*)-**9** to Zn^2+^ at the presence of 1.0 mg/mL hen egg white (HEW) lysozyme in 60% acetonitrile/water. (**a**) Fluorescence (Ex: 280 nm); (**b**) CD; and (**c**) ΔF [52]. Reprinted with permission from Reference [52]. Copyright (2004) American Chemical Society.

A new approach, differential circularly polarized fluorescence excitation (CPE), to metal ion sensing using fluorescence-detected circular dichroic detection was developed, which integrates fluorescence and exciton coupled circular dichroism methods to give better contrast than can be achieved in either of the two parent methods. This approach uses the ΔF (ΔF = F_L_ − F_R_, F_L_, F_R_ = fluorescence with left and right circularly polarized excitation, respectively) [70] component of fluorescence-detected circular dichroism (FDCD) for metal sensing. The contrast in ΔF signals between a sample with both a large quantum yield and a large CD and a sample with both a small quantum yield and a small CD will be much larger than the contrast in either fluorescence or CD signals. The corresponding spectra of one of the sensors employed, compound **9** ((*S*,*S*) form, structure shown in Figure 7), titrated with Zn(II), are shown in Figure 15 [52]. Apparently, measurements in ΔF gave greatly enhanced contrast over other spectroscopic methods. On the coordination chemistry front, stereochemical control and rigidification employed in these sensors ensures improved Zn^2+^/Cu^2+^ selectivity. On the photophysical signal transduction front, the CPE approach has the potential to improve contrast and diminish interference from background fluorescence, such as that from the protein lyzozyme which contains tryptophan (a common source of background fluorescence in cells) (Figure 16).

## 4. Modulating Two Binding Pockets in One Ligand

### 4.1. Modulating Ditoptic Ligands for Zn^2+^

**Figure 17:**
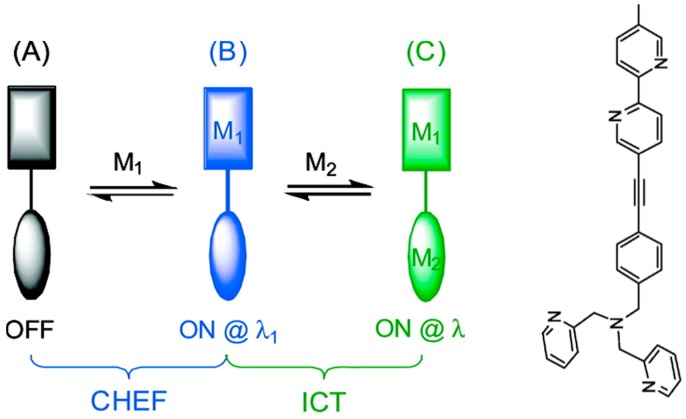
Schematic representation of pyridyl-containing ditoptic sensors (**A**) for both low (**B**) and high concentrations (**C**) of Zn^2+^ with fluorescence enhancement at two different wavelength channels, respectively (**left**); and one of such sensors (**right**). Reprinted with permission from Reference [72]. Copyright (2008) American Chemical Society.

There can be two or more binding pockets in one ionophore. The Zhu group designed a series of fluorescent sensors based on a pyridyl-containing platform bearing two binding pockets with different affinities to Zn^2+^ (Figure 17) [71,72,73,74,75,76,77,78]. Through the modulation of PET, internal charge transfer (ICT), conformation rigidification, and substitution, such ditopic ligands can bind low concentration Zn^2+^ through the high-affinity pocket (bis- or tris-(2-methylpyridyl)amine) to give fluorescence enhancement in one wavelength channel and bind high concentration Zn^2+^ through both the high-affinity and low-affinity(2,2′-bipyridyl) pockets to result in fluorescence enhancement at another wavelength channel. Such sensors have been proven to be useful in live-cell imaging of free Zn^2+^ over a concentration range of six orders of magnitude [79].

### 4.2. Modulating Switchable Binding Pockets in One Ligand

**Figure 18:**
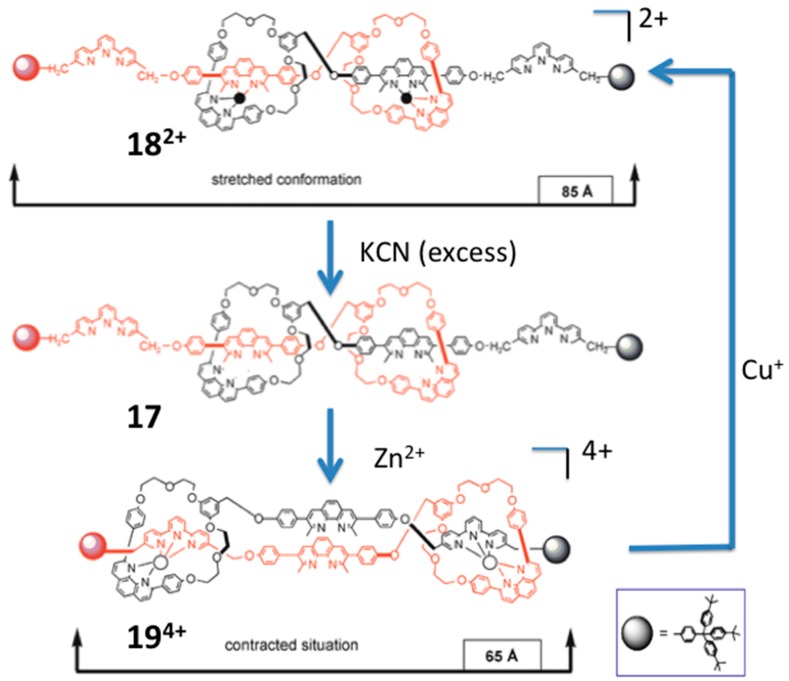
A terpyridyl and phenanthrolyl-containing rotaxane can switchably bind Cu^+^ and Zn^2+^, resulting in extension and contraction, respectively. Adapted with permission from Reference [81]. Copyright (2003) the Royal Society of Chemistry.

Sauvage et al. made a molecular muscle system [80,81] employ a rotaxane dimer **17**: its Cu^+^ complex **18^2+^** is the extension state in which the bidentate phenanthrolinyl of a macrocyle are pulled near to the bidentate phenanthrolinyl in the middle of the molecule by Cu^+^, and its Zn complex **19^4+^** is the contraction state in which the bidentate phenanthrolinyl of the macrocycle is pulled near the tridentate terpyridyl at the two ends of the molecule by Zn^2+^ (Figure 18).

### 4.3. Modulating Switchable Chiroptical Sensors for Metal Oxidation States

Pyridyl and phenanthrolinyl groups were incorporated in catenane [82] and rotaxane [83,84] systems with two binding pockets that can switchably bind copper ions at different oxidation states by turning a bischelating macrocycle containing both bidentate (phenanthroline) and tridentate (terpyridine) moieties around a bidentate (phenanthroline) axel.

Pyridyl groups have also been used in the design of chiral sensors whose coordination chemistry and signal transduction are sensitive to the metal ions’ oxidation states.

**Figure 19:**
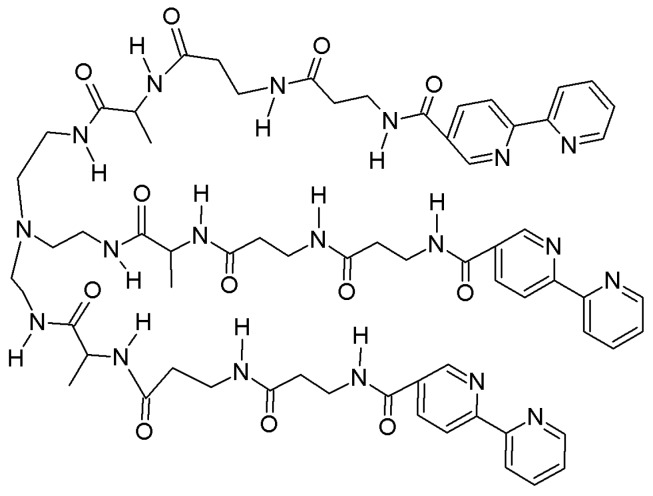
A triple-stranded ligand can switchably bind Fe^2+^ and Fe^3+^ at two different sites, respectively [85].

**Figure 20:**
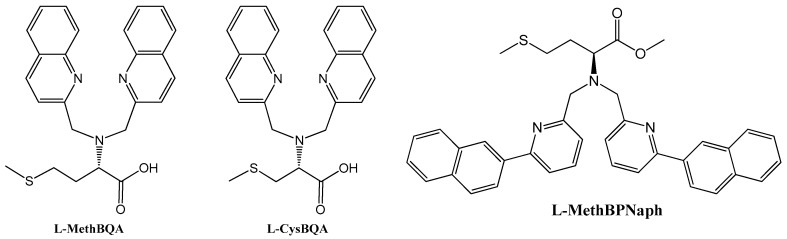
Structures of l-MethBQA, l-CysBQA and a recently synthesized analog l-MethBPNaph.

**Figure 21:**
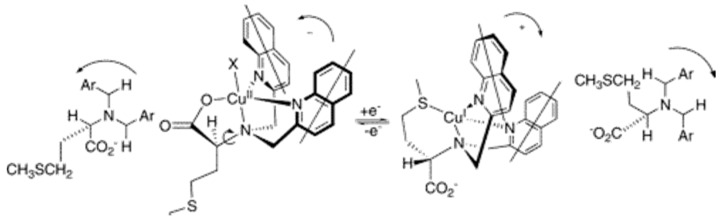
Redox-induced inversion of helicity in copper complexes of l-MethBQA. As a result of the presence of gearing among the three arms of the tripod near the sterically crowded tertiary amine of the ligand, a pivot about a C-N bond results in the inversion of the propeller, giving give opposite exciton-coupled circular dichroism (ECCD) spectra. Reprinted with permission from Reference [90]. Copyright (2006) American Chemical Society.

Shanzer et al. reported such a sensor which employs a triple-stranded system that accommodated a single metal ion in one of two sites, either a “hard” binding cavity bearing three hydroxamate moieties preferable to Fe^3+^ or a “soft” cavity with three bipyridyl moieties preferable to Fe^2+^ (Figure 19) [85]. A split CD spectrum in the UV region was three times more intense for the Fe^2+^ than for Fe^3+^, suggesting exciton interactions involving the bipyridyl groups, which originate from the helical arrangement of each of the three strands. Since switching metal oxidation states can be achieved through redox processes, such processes can be monitored by switchable chiroptical sensors. Chiral tripodal ligands bearing a chiral arm and two achiral 2-methylquinolyl arms can form propeller-like metal complexes whose configuration is dictated by the chiral centers in such ligands as proven in many crystallographic structures in the solid state and ECCD in solution. Such a ligand, *N*,*N*-Bis(2-quinolylmethyl)-l-methionine (l-MethBQA) (Figure 20) [86], derived from the amino acid methionine forms a tetradentate complex with Cu^2+^ involving three nitrogen atoms and a carboxylate. Upon binding Cu^+^, the ligand reorganizes and the sulfide moiety replaces the carboxylate from coordination. Binding Cu^2+^ and Cu^+^ produce opposite helical orientation of the two quinolyl moieties, resulting in mirror images in the ECCD spectra for the Cu^+^ vs. Cu^2+^ complexes. Other such tripodal derivatives of methioninol and *S*-methylcysteine can also sense the oxidation states of copper ions following the same mechanism (Figure 21) [87,88,89,90].

### 4.4. Modulating Switchable Chiroptical Sensors for Multiple Metal Ions

**Figure 22:**
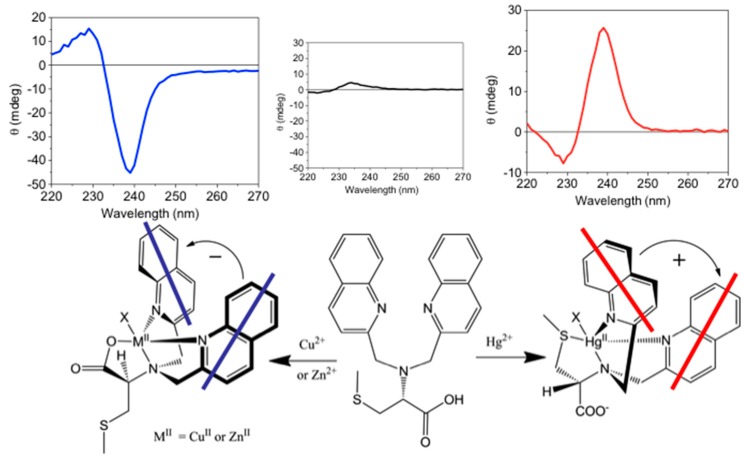
l-CysBQA complexes with Cu^2+^/Zn^2+^ and Hg^2+^. The chiral center of the amino acid dictates the orientation of the quinoline chromophores via a gearing mechanism as illustrated. The transition dipoles in the quinolines in the two complexes invert in the sense of absolute orientation and therefore give opposite ECCD spectra. Adapted with permission from Reference [61]. Copyright (2011) Wiley.

l-MehtBQA and a similar compound *N*,*N*-Bis(2-quinolylmethyl)-l-*S*-methylcysteine (l-CysBQA) (Figure 20), were found to be multimode switchable chiroptical fluorescent sensors for multiple ions including but not limited to Hg^2+^, Cu^2+,^ and Zn^2+^ [61]. Quinolyl groups serve as the fluorophore and possess nitrogen lone pairs capable of chelating metal ions. Upon exposure to Hg^2+^ or Zn^2+^ these sensors show signal enhancement in fluorescence in 30:70 acetonitrile/water. It is likely that the interaction between the Hg^2+^ ion and the fluorophore is limited by the covalently binding S atom, disrupting the heavy atom effect and resulting in fluorescence enhancement [60]. However, Cu^2+^ quenches their fluorescence. l-CysBQA complexes with Hg^2+^, giving rise to an exciton-coupled circular dichroism spectrum with a positive couplet (a positive Cotton effect at a longer wavelength followed by a negative Cotton effect at a shorter wavelength). However, Cu^2+^ or Zn^2+^ complexation produces a negative ECCD couplet (Figure 22). This remarkable differentiation of Hg^2+^ from Cu^2+^ and Zn^2+^ stems from the different structures of the CD active products. The Cu^2+^ ion binds with the tertiary amine, the two quinolones, and the carboxylate moieties of the ligand. The two quinoline groups form a propeller whose orientation is dictated by the stereocenter of the *S*-methyl cysteine arm. However, Hg^2+^ prefers coordination by the sulfur atom. As shown in Figure 22, for the sulfide to bind to the metal center, the amino acid arm must pivot about the C-N bond, which inverts the orientation of the quinoline moieties, leading to an exciton coupled CD with the opposite sign. Although crystals of l-CysBQA complexes with these metal ions are not available, Zn^2+^ and Cu^2+^ coordinate with the carboxylate instead of the sulfide in the crystals of their complexes with l-MethBQA and other similar compounds [88,89,90]. In solution, the soft Cu^+^ ion coordinates with the sulfur atoms of such ligands in solution as demonstrated by NMR data and other evidence [88,91], which indicates that Hg^2+^ should coordinate the sulfur atom since it is also soft.

**Figure 23:**
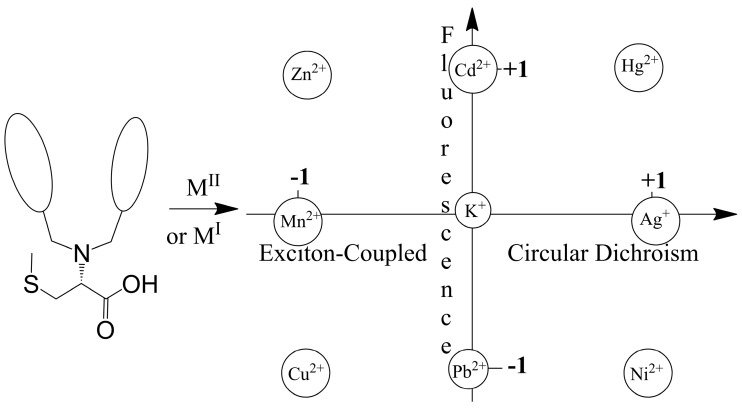
Chiroptically enhanced fluorescence detection and differentiation of different metal ions by l-CysBQA through pattern recognition. Adapted with permission from Reference [61]. Copyright (2011) Wiley.

The design of such sensitive and selective chiroptical fluorescent sensors for metal ions includes innovations on both coordination chemistry and signal transduction. Take the above mentioned mercury sensors for example. On the coordination chemistry front, ionophores are equipped with two sets of coordination “teeth”, switchable by exposure to different metal ions. One set of “teeth” offers high affinity for Hg^2+^, determining the sensitivity of the probe; the other set preferably binds other metal ions. On the photophysical signal transduction front, the binding between Hg^2+^ and its preferred set of “teeth” leads to fluorescence enhancement and a positive exciton coupled CD by design; and the coordination of the other set of “teeth” with other metal ions, such as Zn^2+^ and Cu^2+^, triggers a fluorescence change (enhanced or quenched) and a negative exciton coupled CD. Other metal ions produce other combinations of fluorescence and exciton coupled CD. In this way, metal ion sensing by such chiroptical fluorescent sensors through both fluorescence and anisotropic absorption distinguishes (Figure 23), for example, Hg^2+^ (enhanced fluorescence with strong positive exciton coupled CD (ECCD)), Zn^2+^ (enhanced fluorescence and strong negative ECCD), Cu^2+^ (strong negative ECCD but quenched fluorescence), Ni^2+^ (strong positive exciton coupled CD but quenched fluorescence), Pb^2+^ (quenched fluorescence but no ECCD), Cd^2+^ (enhanced fluorescence but no ECCD). More ions such as Cd^2+^(enhanced fluorescence and no exciton coupled CD), Ag^+^ (no fluorescence change and strong positive exciton coupled CD) and alkali metal ions (no change in fluorescence or CD) can be added to the list [92]. l-MethBQA offers similar advantages. These results further illustrate that recognition involving both isotropic and anisotropic detection tools may be utilized to maximize the information transmitted by a single sensor molecule [46].

Interestingly, a pyridyl analog l-MethBPNaph (Figure 20) shows fluorescence enhancement as well as significant spectral red-shift in emission upon exposure to HgCl_2_ [93]. However, its CD spectrum does not changed upon addition of HgCl_2_, ZnCl_2_, CuCl_2_, Zn(ClO_4_)_2_, Cu(ClO_4_)_2_, Ni(ClO_4_)_2_ or Pb(ClO_4_)_2_.

Aside from metal ions, pyridyl containing systems have been used as chiroptical sensors for anions [64,94] and other species, some of which have been recently reviewed [93,95,96]. Recently, a pyridyl-containing homochiral, square-shaped, *D*_2_ symmetrical metal-linked macrocycle has been shown to be a selective chiroptical and electrochemical sensor for ferrocene in the presence of other species [97].

## 5. Conclusions

Pyridyl- or quinolyl-containing compound are excellent platforms to build selective ionophores and sensors for metal ions. Steric and stereochemical effects can be used to modulate such ionophores and sensors in terms of coordination chemistry and/or signal transduction. On the coordination chemistry front, a ligand’s affinity and selectivity toward metal ions can be systemically fine-tuned or switched through modification of ligand structures by introducing/removing steric crowding or adding chiral handles, which creates a high degree of preorganization for the specific metal ion and/or a high degree of ‘disorganization’ or mismatch for competing metal ions. On the photophysical signal transduction front, steric effect can be used to engineer “turn-on” fluorescent sensors for heavy metal ions and control the extent of chelation induced enhancement of fluorescence. Stereochemical modification can bring additional spectroscopic information such as chiroptical signals into the signal transduction part of sensing, which is capable of making sensing events more specific. Sensing strategies employing both isotropic and anisotropic absorption signals from a single chiral sensory molecule provide not only detection but also differentiation of multiple analytes.
